# Resveratrol-Loaded Lipid-Core Nanocapsules Modulate Acute Lung Inflammation and Oxidative Imbalance Induced by LPS in Mice

**DOI:** 10.3390/pharmaceutics13050683

**Published:** 2021-05-10

**Authors:** Maria Talita Pacheco de Oliveira, Diego de Sá Coutinho, Sílvia Stanisçuaski Guterres, Adriana Raffin Pohlmann, Patrícia Machado Rodrigues e Silva, Marco Aurélio Martins, Andressa Bernardi

**Affiliations:** 1 Laboratory of Inflammation, Oswaldo Cruz Institute, Oswaldo Cruz Foundation, Rio de Janeiro 21040-360, Brazil; talipacheco@gmail.com (M.T.P.d.O.); patsilva1910@gmail.com (P.M.R.eS.); mmartins@ioc.fiocruz.br (M.A.M.); 2 Pharmaceutical Sciences Post-Graduation Program, College of Pharmacy, Federal University of Rio Grande do Sul, Porto Alegre 90035-003, Brazil; silvia.guterres@ufrgs.br (S.S.G.); adriana.pohlmann@ufrgs.br (A.R.P.); 3 Department of Organic Chemistry, Institute of Chemistry, Federal University of Rio Grande do Sul, Porto Alegre 90035-003, Brazil

**Keywords:** acute lung injury, acute respiratory distress syndrome, inflammation, LPS, resveratrol, nanocarriers

## Abstract

Acute lung injury (ALI) and acute respiratory distress syndrome (ARDS) are inflammatory and oxidative imbalance lung conditions with no successful pharmacological therapy and a high mortality rate. Resveratrol (RSV) is a plant-derived stilbene that presents anti-inflammatory and antioxidant effects. However, its therapeutic application remains limited due to its poor bioavailability, which can be solved by the use of nanocarriers. Previously, we demonstrated that nanoencapsulated RSV (RSV-LNC) pre-treatment, performed 4 h before lipopolysaccharide (LPS) stimulation in mice, increased its anti-inflammatory properties. In this study, we evaluated the anti-inflammatory and antioxidant effects, and lung distribution of RSV-LNCs administered therapeutically (6 h post LPS exposure) in a lung injury mouse model. The results showed that RSV-LNCs posttreatment improved lung function and diminished pulmonary inflammation. Moreover, RSV-LNC treatment enhanced the antioxidant catalase level together with a decrease in the oxidative biomarker in mouse lungs, which was accompanied by an increase in pulmonary Nrf2 antioxidant expression. Finally, the presence of RSV in lung tissue was significantly detected when mice received RSV-LNCs but not when they received RSV in its free form. Together, our results confirm that RSV nanoencapsulation promotes an increase in RSV bioavailability, enhancing its therapeutic effects in an LPS-induced lung injury model.

## 1. Introduction

Acute lung injury (ALI) and the most severe form of acute respiratory distress syndrome (ARDS) represent a combination of clinical signs characterized by severe inflammation and diffuse alveolar damage, which lead to alveolar edema and reduction in tissue oxygenation [1,2]. Together, it results in a high mortality rate with a reduction in survivors’ quality of life [3]. Currently, no effective pharmacological therapy is available for preventing or managing ALI/ARDS, and supportive care focuses on increasing oxygen delivery and avoiding further injury [1,4]. Even with inflammation, oxidative stress plays a central role in disease progression. Oxidative damage is characterized by an imbalance between reactive oxygen species (ROS) and reactive nitrogen species (RNS) production and antioxidant defense levels [5,6]. In ALI/ARDS pathology, immune cells, such as neutrophils and macrophages, release a large amount of cytokines and ROS [7]. Secreted ROS can lead to direct damage to DNA cells, oxidation of proteins, induction of protease release, and inactivation of antioxidant and antiprotease enzymes. These events enhance the expression of proinflammatory genes, inducing lipid peroxidation, and perpetuate lung damage [5]. Therefore, the use of anti-inflammatory/antioxidant agents represents a promising therapy to treat the disease [7].

Medicinal plants and their secondary metabolites have been used for the treatment of inflammation since ancient times based on widespread use by successive generations [8]. Among natural bioactive molecules, resveratrol (RSV) or trans-3,4,5-trihydroxystilbene is a natural polyphenol synthesized by fruits, such as blueberries, red grapes, and peanuts, as a response to unfavorable or stressful conditions, damage, and mechanical injury [9,10]. RSV presents marked anti-inflammatory and antioxidant activities and can interact with multiple molecular targets, presenting benefits in numerous diseases [11]. Several studies have focused on RSV for the treatment of ALI/ARDS disorders in different lung injury models [12,13,14]. However, the poor bioavailability limits the therapeutic activity of RSV given its low solubility, reduced stability, short half-life, intense metabolism, and high rate of excretion [15,16].

Many efforts have been made to minimize the poor bioavailability of RSV, including the development of nanoformulations aiming to improve delivery [17,18]. The use of nanocarriers provides drug protection against biological barriers and degradation, allows the stable dispersion of poorly soluble molecular agents, and enables compound vectorization to tissues [19]. Different types of nanomaterials have been used as targeted carriers, including poly(ε-caprolactone) (PCL) lipid-core nanocapsules (LNCs) [20]. PCL-LNCs, which are composed of an oil nucleus revested by a PCL polymeric schedule, have a high encapsulation capacity of lipophilic substances and are considered a promising device in drug delivery [21,22]. The use of PCL as a polymer is appropriate once it presents biodegradability and biocompatibility capacity, which makes it an up-and-coming drug carrier with high potential for therapeutic applications [22].

Our workgroup has recently demonstrated that pretreatment with RSV incorporated into polymeric lipid-core nanocapsules (RSV-LNCs) 4 h before lipopolysaccharide (LPS) provocation in mice improved lung function and reduced pulmonary tissue damage and inflammatory cell infiltration at lower doses compared to free RSV [23]. In this study, we evaluated the anti-inflammatory activity of RSV in a therapeutic protocol, with the treatment being performed 6 h after LPS exposure. We also evaluated the impact of RSV nanoencapsulation posttreatment on oxidative imbalance induced by LPS and on mouse lung biodistribution.

## 2. Materials and Methods

### 2.1. Preparation of RSV-LNCs

The polymeric nanocapsules containing resveratrol (RSV-LNCs) preparation were carried out according to Oliveira et al. and Frozza et al., employing the interfacial polymer deposition method, and this study description method partly reproduces their wording [23,24]. To obtain an organic solution, the following reagents were dissolved in 27 mL of acetone at 40 °C: 0.010 g of resveratrol, 0.100 g of poly(ε-caprolactone), 0.33 mL of capric/caprylic triglyceride, and 0.038 g of sorbitan monostearate. The aqueous phase consisted of 0.038 g of polysorbate 80 in 53 mL of ultrapure water (Milli-Q^®^). The protocol for obtaining the nanocapsules was carried out at room temperature by the injection of the organic solution in the aqueous phase under magnetic stirring for 10 min. The acetone was then evaporated, concentrated under reduced pressure, and adjusted to a final volume of 10 mL. The same procedure was performed without the addition of RSV to produce drug-unloaded nanocapsules (ULNCs) for the control vehicle solution.

### 2.2. Physicochemical Characterization of RSV-LNCs

Measurements were performed using 3 different batches for each formulation in triplicate. For each formulation batch, the volume-weighted mean diameter (D_4,3_) and the polydispersity index (span) were verified using the Dispersion Technology Software–DTS Nano–Version 5.02 software (Malvern Instruments Ltd., Malvern, UK). Samples were diluted with ultrapure water or NaCl aqueous solution (0.01 mol/L) to assess the z-average (mean diameters), polydispersity index, and zeta potentials by dynamic light scattering using Zetasizer^®^ nano-ZS ZEN 3600 model (Nanoseries, Malvern, UK). The pH was determined by a potentiometer (Highmed^®^, São Paulo, Brazil). To determine the total RSV concentration, using high-performance liquid chromatography (HPLC) method at 306 nm, the RSV-LNC formulations (100 µL) were dissolved in 10 mL of acetonitrile and then filtered (Millipore 0.45 µm). This description partly reproduces the wording from de Oliveira et al. For more details on the preparation of the formulations, please check the information [23].

### 2.3. Animals

Male A/J mice weighing 18–20 g (Laboratory Animal Breeding Center of the Oswaldo Cruz Foundation—FIOCRUZ, Rio de Janeiro, Brazil) were kept in the animal housing facilities (maximum of 5 mice per cage). The animals had free access to food and water, a 12-h light-dark cycle, and room air conditioning (22–25 °C). The in vivo trials were evaluated and approved by the Animal Ethics Committee of the Oswaldo Cruz Foundation on 17 February 2016 (Protocol L-006/2016; Ethics Committee on the Use of Animals-FIOCRUZ).

### 2.4. Murine Model of LPS-Induced Lung Injury and Treatment Protocol

This study uses the method previously described in D’Almeida with adaptations in the treatment protocol, and the method description partly reproduces their wording [25]. Animals anesthetized with isoflurane aerosol and constant O_2_ flow (Cristália^®^, Itapira, Brazil) were stimulated intranasally with 25 µg of LPS (Sigma-Aldrich, St. Louis, MO, USA) prepared in 25 µL of sterile saline. The control group received the same amount of sterile saline. RSV, RSV-LNC, or dexamethasone (Dexa) treatment was administered orally (5 mg/kg) 6 h after LPS instillation. The analyses were performed 24 h after the LPS challenge.

### 2.5. Assessment of Pulmonary Function and Airway Hyperreactivity

As previously described in Coutinho et al. [26] with slight modifications, airway hyperreactivity (AHR) was calculated from pulmonary elastance data, after aerosolization with methacholine, according to the Buxco FinePoint R/C system (Buxco Electronics, Sharon, CT, USA). For that, animals anesthetized with 60 mg/kg of sodium pentobarbital (Cristália, Brazil) were curarized with pancuronium bromide (Pavulon^®^, 1 mg/kg) (Sigma-Aldrich, USA), tracheostomized and then submitted to mechanical ventilation. After system stabilization (5 min), increasing concentrations of methacholine (3, 9, and 27 mg/mL) were aerosolized for 5 min each to assess airway responsiveness. The elastance data are represented as the area under the curve for the methacholine concentration mean values.

### 2.6. Cell Recovery from the Airway Lumen

This method description partly reproduces Oliveira et al.’s wording [23]. Briefly, after overdosing with 500 mg/kg of sodium pentobarbital, bronchoalveolar lavage was performed with PBS + EDTA 10 mM (Sigma-Aldrich, USA), using a polyethylene cannula inserted in the animal’s trachea. Soon after, the bronchoalveolar lavage fluid (BALF) was centrifuged and the cell pellets were resuspended in the same solution for counting total leukocytes with the help of a Neubauer chamber. For cell morphological differentiation, Cytospin slides were prepared with May–Grunwald–Giemsa staining.

### 2.7. Myeloperoxidase Activity Assay

The assay to detect the presence of myeloperoxidase (MPO) was carried out as reported before [23]. After perfusing, fragments of right lungs were removed, homogenized (on ice, in 1 mL of Hank’s solution), and centrifuged. The supernatant was discarded, and the pellet resuspended in both hypo and hypertonic sodium chloride solutions, then centrifuged again. To the formed pellet was added 1 mL of hexadecyltrimethylammonium bromide (HTAB) and recentrifuged. Next, in 96-well plates, the supernatant (50 μL) was added together with HTAB (50 μL) and orthodianisidine (50 μL) for 15 min at 37 °C, and then 50 μL of H_2_O_2_ was added to each well. After 10 min, sodium azide (50 μL) was added. The samples underwent analysis in a spectrophotometer (460 nm), and the result was adjusted by total protein.

### 2.8. Inflammatory Mediator Quantification

According to [26], right lungs were perfused, removed, and homogenized (on ice) in buffer solution (PBS, 1 mL) containing Triton X-100 (0.05%) and protease inhibitor cocktail (Complete^®^; Hoffmann-La Roche Ltd., Basel, Switzerland). Supernatants were recovered after centrifugation for immunoassay according to the manufacturer’s instructions (DuoSet^®^; R&D Systems, Minneapolis, MN, USA). Cytokine levels were normalized by the total protein content in the samples and expressed as cytokine picograms per milligram of protein.

### 2.9. Pulmonary Tissue Analysis

The left lung lobe was removed, after perfusion, and quickly placed in a Millonig buffer solution (pH = 7.4) with 4% paraformaldehyde to preserve tissue characteristics. After routine histological procedures, sections of lung fragments were stained with H and E and scanned for analysis using the Pannoramic Viewer program (3DHISTECH Ltd., Budapest, Hungary). The structure of the tissue was evaluated, according to [23], considering the following criteria: the presence of leukocytes, alveolar congestion and thickness of the alveolar wall. The following numerical classification system (0–4) was used: 4 = very severe damage, 3 = severe damage, 2 = moderate damage, 1 = minor damage, and 0 = no damage.

### 2.10. Oxidative Imbalance Analysis

Fragments of mouse right lungs were perfused, removed, and homogenized on ice in 500 μL of potassium phosphate + EDTA buffer (KPE) (pH 7.5), and then the samples were centrifuged. The supernatant was used to determine the malondialdehyde (MDA) levels and catalase activity. Catalase activity was measured by the conversion of hydrogen peroxide (H_2_O_2_) to H_2_O and O_2._ As described by [27] with slight modifications, 1 part of the lung homogenate samples was added to 99 parts of the solution containing 0.05 M phosphate buffer (50 mL) and hydrogen peroxide (0.3 mL) at pH 7.0. Using a spectrophotometer (240 nm), absorbance measurements were acquired after 0, 30, and 60 s. The result represents catalase units per milligram of protein. In turn, lipid peroxidation was estimated by the presence of MDA [28]. Briefly, a mixture of sample and 10% trichloroacetic acid (1:1) was centrifuged for 15 min (3600× *g*) at 4 °C, then equal parts of supernatant and thiobarbituric acid were heated for 10 min at 95 °C. The final absorbance was recorded by a spectrophotometer (532 nm). Data are expressed as nM per mg of protein.

### 2.11. Quantification of RSV in the Lung Tissue

To determine RSV levels in lung tissue, a reverse phase HPLC analysis was performed. In summary, animals received an overdose of anesthetic (sodium pentobarbital) after 24 h of treatment. Shortly thereafter, the perfused lung tissues were quickly removed and weighed. To extract the RSV, acetonitrile (3 mL per lung) was added, and the tissue was manually macerated. Then, the resulting mixture was centrifuged (3000× *g* for 10 min) and the filtered supernatant (0.45 μM; Merck Millipore) was injected (20 μL) into the liquid chromatography system. The system used had a Shim-pack CLC-C8 (M) column (150 mm, 4.6 mm, 5 m, Shimadzu Corporation, Japan) with a guard column, a UV-Vis detector, pump, and S200 Perkin auto-injector -Elmer (PerkinElmer Instruments, Norwalk, CT, USA). The mobile phase consisted of HPLC grade acetonitrile and ultrapure water (40:60 *v/v*). The pH was corrected with 10% (*v/v*) orthophosphoric acid to achieve 3.0 ± 0.5. RSV retention occurred in 3.45 min, considering the isocratic flow of the mobile phase of 1.2 mL/min. In this assay, linearity, inter and intra-day variability, selectivity, and accuracy were considered to validate the method. Linear calibration curves for RSV were detected between 2.5 and 17.5 μg/mL (r > 0.9999), with 2.00 μg/mL being the limit of quantification. Using numerical integration, the RSV concentration was determined by calculating the peak area ratio of tissue samples from animals treated with the corresponding concentration of RSV in acetonitrile.

### 2.12. Western Blot Analysis

As described in [29], with few modifications, fragments of the right lung of mice were perfused, removed, and homogenized on ice in a buffer solution enriched with protease and phosphatase inhibitors (1:100; Thermo Fisher Scientific, Waltham, MA, USA). After centrifugation (13,000× *g* for 10 min at 4 °C), the supernatant was recovered for analysis. The BCA method was performed to quantify the total protein concentration. The samples, mixed with β-mercaptoethanol (final concentration of 5%), were heated to 100 °C for 5 min for protein denaturation. Equal amounts of protein (40 μg) were separated on SDS-PAGE gel (10–12%) and electrotransferred to nitrocellulose membranes through a semi-wet transfer system (Trans-Blot^®^ SD; Bio-Rad, Hercules, CA, USA). To avoid unspecific binding, the membranes were blocked by incubation with nonfat dry milk (5%) in TBS for 1 h at room temperature. After washing, the membranes were incubated overnight (4 °C) with the primary antibodies against ERK1/2 proteins (1:1000; Cell Signaling, Danvers, MA, USA), p38 (1:1000; Cell Signaling, Danvers, MA, USA), AKT (1:1000; R&D, Minneapolis, MN, USA) and Nrf2 (1:1000; ABCAM. Cambridge, UK). Then, the membranes were incubated with their respective secondary antibodies attached to horseradish peroxidase at a ratio of 1:1000 (1 h, room temperature). Anti-β-actin (1:1000; Abcam, Cambridge, UK), used as a control, was incubated overnight (4 °C) and then incubated with HRP goat anti-mouse (1:10,000; R&D Systems) for 1 h. The proteins were visualized using a chemiluminescence detection system in X-ray films (Kodak™; PerkinElmer), which were then digitized for bands evaluation by ImageJ software. The results represent the ratio of phosphorylated protein to total protein or total protein to β-actin protein.

### 2.13. Statistical Analysis

Data were analyzed by one-way analysis of variance (ANOVA) and then by a Newman–Keuls post hoc test (GraphPad Software, 5.01, La Jolla, CA, USA). The results are presented as the mean ± standard error of the mean (SEM). *p*-values < 0.05 were considered statistically significant.

## 3. Results

### 3.1. Physicochemical Characteristics of Polymeric Nanocapsules

The LNC formulations were prepared and did not require subsequent purification. Both nanocapsule formulations (RSV-LNCs and LNCs) showed opalescent white color, a homogeneous appearance (without precipitate formation), monomodal size distributions, and a polydispersity index of 0.16 ± 0.03 nm (RSV-LNC) and 0.17 ± 0.02 (LNC). The average particle diameters were 247 ± 9 nm and 254 ± 10 nm for RSV-LNC and LNC, respectively. The zeta potential was −12.8 ± 1.5 mV for RSV-LNC and −10.5 mV ± 2 mV for LNC, with pH values of 5.4 ± 0.3 (RSV-LNC) and 5.6 ± 0.1 (LNC). The encapsulation efficiency was approximately 100%, with RSV content of 0.998 ± 0.010 mg/mL.

### 3.2. Effect of RSV-LNCs on Airway Hyperreactivity (AHR) and Leukocyte Infiltration

**Figure 1:**
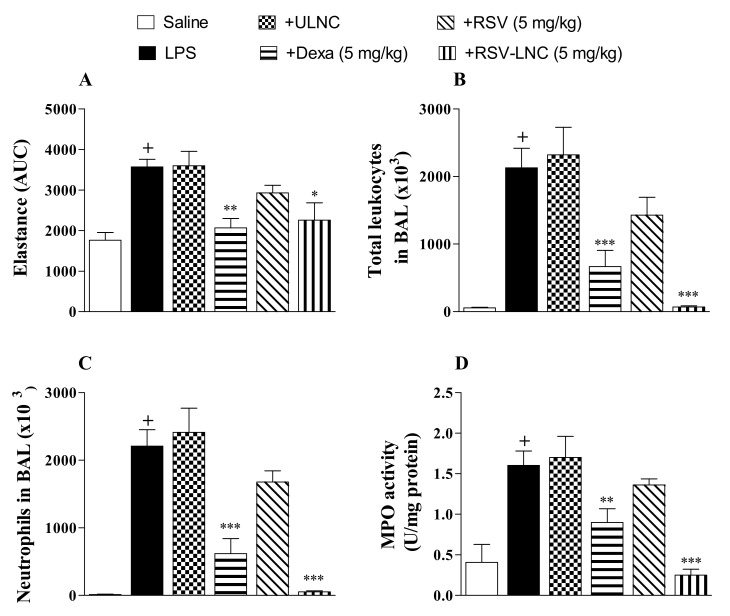
Effect of treatment with RSV or RSV-LNCs on AHR and pulmonary inflammation induced by LPS instillation. Mice were orally treated with ULNCs, Dexa, RSV, or RSV-LNCs (5 mg/kg) 6 h after intranasal exposure to LPS (25 μg/25 μL). (**A**) AHR was measured 24 h after LPS instillation and is represented as the area under the curve (AUC) obtained from the mean values of methacholine concentration aerosolization (3–27 mg/mL). After AHR measurement, cells were collected by bronchoalveolar lavage for (**B**) total leukocyte and (**C**) neutrophil counts. Then, lungs were perfused and collected to measure (**D**) MPO activity. Data are expressed as the mean ± SEM from 5–8 animals. + *p* < 0.05 compared with the saline group; * *p* < 0.05, ** *p* < 0.01 and *** *p* < 0.001 compared to the LPS group.

Mice were instilled with LPS (25 µg/µL) to compare the effect of therapeutic treatment with RSV and RSV-LNCs on lung function changes and pulmonary inflammation. After 6 h, at which time inflammation was already installed, the animals were treated with the formulations. The area under the curve data showed a significant increase in lung elastance in the LPS-exposed group compared to the saline study group 24 h after LPS instillation (Figure 1A). Intense leukocyte influx into the airway lumen was also observed (Figure 1B). The inflammatory response was marked by the presence of neutrophils in both bronchoalveolar fluid (Figure 1C) and lung tissue as demonstrated by the increase in MPO pulmonary levels (Figure 1D). Oral posttreatment with the standard drug dexamethasone or with RSV-LNCs (5 mg/kg), but not with UNLC vehicle or RSV, at the same dosage, inhibited the increase in pulmonary elastance and abolished lung leukocyte accumulation induced by LPS provocation.

### 3.3. Effect of RSV-LNCs on Pulmonary Structural Damage

**Figure 2:**
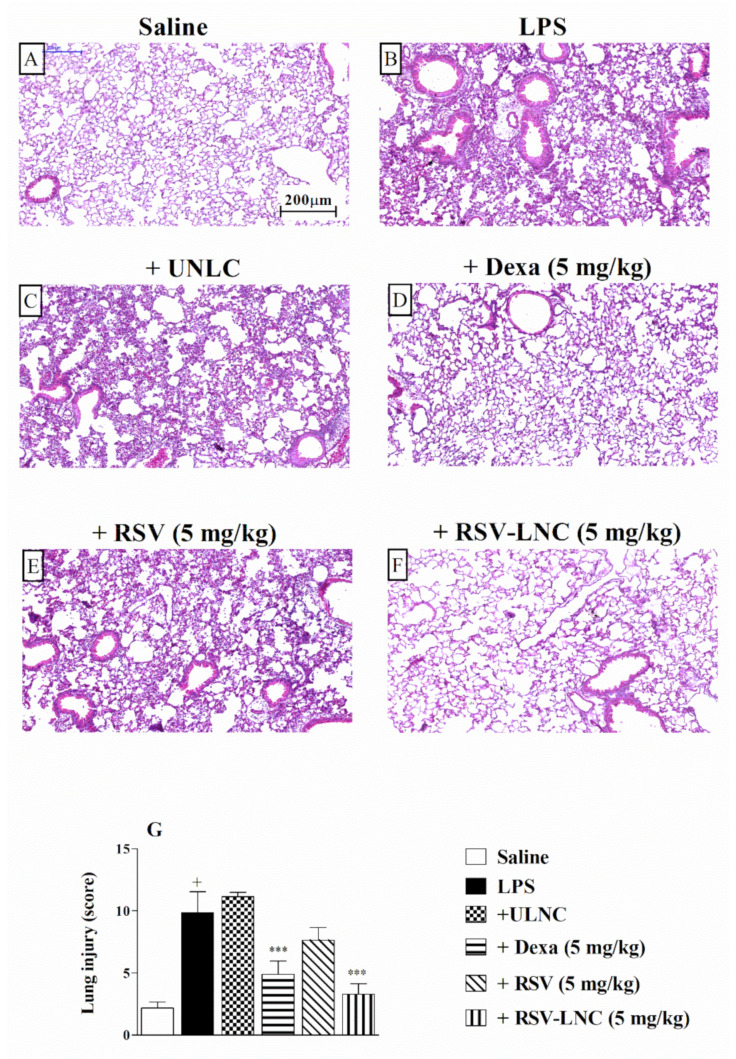
Effect of treatment with RSV or RSV-LNCs on lung damage induced by LPS instillation. Mice were orally treated with ULNCs, Dexa, RSV or RSV-LNCs (5 mg/kg) 6 h after intranasal exposure to LPS (25 μg/25 μL). The lungs were perfused, and the left lobe was collected in formalin 24 h after LPS instillation. Lung sections embedded in paraffin were obtained by routine methods and stained with H and E. (**A**) Saline, (**B**) LPS, (**C**) +UNLC, (**D**) +Dexa (5 mg/kg), (**E**) +RSV (5 mg/kg) and (**F**) +RSV-LNC (5 mg/kg) microscopic images of H and E stained tissue sections. (**G**) Histological score analysis of lung inflammation. Data are expressed as the mean ± SEM from 6–8 animals. + *p* < 0.05 compared with the saline group; *** *p* > 0.001 compared to the LPS group.

The histological analysis of lung tissue demonstrated that LPS stimulation induced important changes in the structure of the parenchyma, including pulmonary congestion (alveoli with red blood cells) and increased thickness of the alveolar walls, when compared to the lungs of healthy animals. (Figure 2A,B, respectively). Treatment with 5 mg/kg dexamethasone (Figure 2D) or RSV-LNCs (Figure 2F) inhibited lung damage. The UNLC or free RSV groups (Figure 2C,E) showed no differences compared to the group that received only LPS. Semiquantitative analysis of histological sections stained with H and E, shown in Figure 2G, confirmed these findings.

### 3.4. Effect of RSV-LNCs on Inflammatory Mediator Levels in Lung Tissue

**Figure 3:**
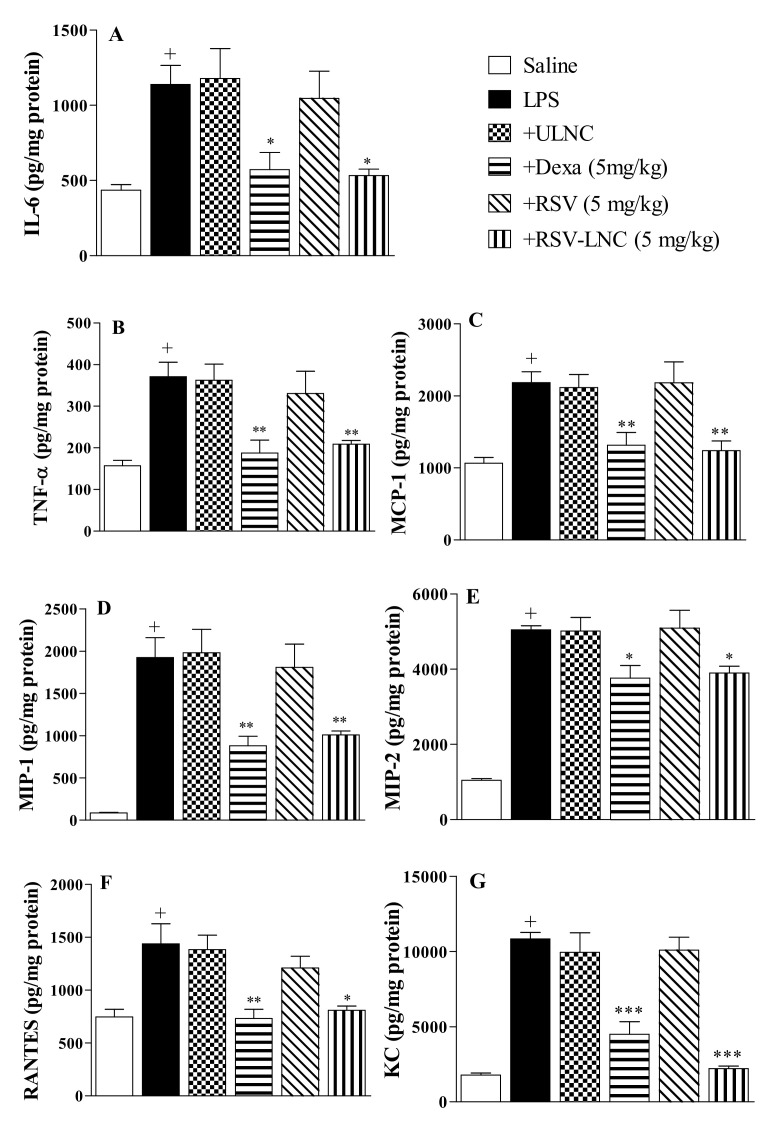
Effect of treatment with RSV or RSV-LNCs on pulmonary cytokine levels induced by LPS instillation. Mice were orally treated with ULNCs, Dexa, RSV or RSV-LNCs (5 mg/kg) 6 h after intranasal exposure to LPS (25 μg/25 μL). After 24 h of LPS instillation, fragments of right lungs were perfused and collected to measure (**A**) IL-6, (**B**) TNF-α, (**C**) MCP-1, (**D**) MIP-1, (**E**) MIP-2, (**F**) RANTES and (**G**) KC levels by ELISA. Data are expressed as the mean ± SEM from 6–8 animals. + *p* < 0.05 compared with the saline group; * *p* < 0.05; ** *p* > 0.01 and *** *p* > 0.001 compared to the LPS group.

The results showed an increase in IL-6 (Figure 3A), TNF-α (Figure 3B), MCP-1 (Figure 3C), MIP-1α (Figure 3D), MIP-2 (Figure 3E), RANTES (Figure 3F), and KC (Figure 3G) levels in lung samples 24 h after LPS exposure. Posttreatment with dexamethasone or RSV-LNCs significantly inhibited the amount of all pro-inflammatory cytokines evaluated. However, no change was observed in the animals treated with ULNCs or RSV compared to the LPS group. In parallel, we assessed whether the lower levels of pro-inflammatory cytokines in the lung tissue, promoted by treatment with RSV-LNCs, were correlated with an increase in the anti-inflammatory cytokine IL-10. Nevertheless, it was not possible to obtain a statistical difference between the groups when measuring the levels of IL-10 in the lung tissue (data not shown).

### 3.5. Molecular Mechanisms Underlying the Anti-Inflammatory Effects of RSV

**Figure 4:**
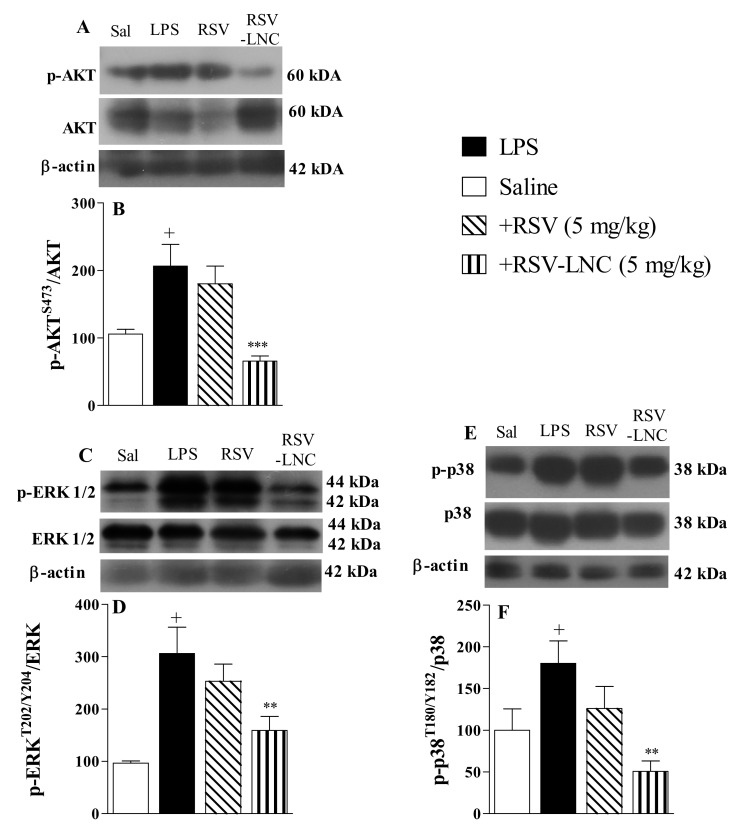
Effect of treatment with RSV or RSV-LNCs on pro-inflammatory molecular pathways underlying lung injury induced by LPS instillation. Mice were orally treated with ULNCs, Dexa, RSV or RSV-LNCs (5 mg/kg) 6 h after intranasal exposure to LPS (25 μg/25 μL). After 24 h of LPS instillation, fragments of right lungs were perfused and collected for pro-inflammatory protein expression quantification. Representative Western blot images of phosphorylation and total (**A**) AKT, (**C**) ERK1/2 and (**E**) p38 and densitometric values of (**B**) AKT and (**D**) ERK1/2 and (**F**) p38 normalized to their respective controls that were not exposed to LPS (control bar; 100%). Data are expressed as the mean ± SEM from 5–9 animals. + *p* < 0.05 compared to saline; ** *p* < 0.01 and *** *p* < 0.001 compared to LPS group.

The results show that LPS stimulation induced an increase in the phosphorylation of Akt (Figure 4A), ERK (Figure 4C), and p38 proteins (Figure 4E) in lung tissue from untreated mice. Densitometric quantification showed that treatment with RSV-LNCs (5 mg/kg) reduced phosphorylation without modifying the total levels of Akt (Figure 4B), ERK (Figure 4D), and p38 proteins (Figure 4F). However, treatment with free RSV at the same dosage failed to reduce the levels of these proteins.

### 3.6. Effect of RSV-LNCs on Oxidative Imbalance

**Figure 5:**
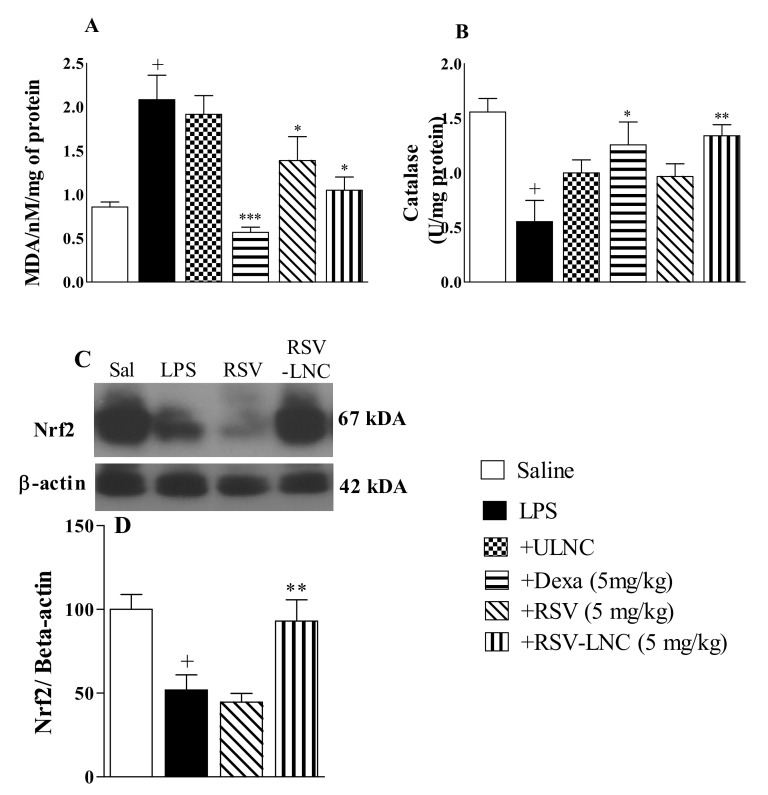
Effect of treatment with RSV or RSV-LNCs on oxidative imbalance induced by LPS instillation. Mice were orally treated with ULNCs, Dexa, RSV or RSV-LNCs (5 mg/kg) 6 h after intranasal exposure to LPS (25 μg/25 μL). After 24 h of LPS instillation, fragments of right lungs were perfused and collected for analysis of (**A**) malondialdehyde, (**B**) catalase enzyme levels and posterior quantification of Nrf2 expression. (**C**) Representative western blot images of Nrf2 and (**D**) densitometric values obtained for β-actin and Nrf2 quantification, normalized to saline control (control bar; 100%). Data are expressed as the mean ± SEM from 5–8 animals. + *p* < 0.05, compared to saline; * *p* < 0.001; ** *p* < 0.05 and *** *p* < 0.0001, compared to the LPS group.

As shown in Figure 5, the LPS inflammatory stimulus was accompanied by an increase in the lipid peroxidation biomarker MDA (Figure 5A) and a decline in catalase antioxidant enzyme levels (Figure 5B) in mouse lungs compared to the saline group. Treatment with both RSV-LNCs and RSV (5 mg/kg) significantly reduced MDA levels, but only treatment with RSV-LNCs restored the levels of catalase in lung tissue samples. Treatment with dexamethasone (5 mg/kg) prevented both of these alterations. No change was observed in mice treated with ULNCs compared to the LPS nontreated group. Moreover, LPS stimulation also reduced Nrf2 expression, which was reversed in mice treated with RSV-LNCs (5 mg/kg) but not with RSV (Figure 5C).

### 3.7. RSV Quantification in Lung Tissue

**Figure 6:**
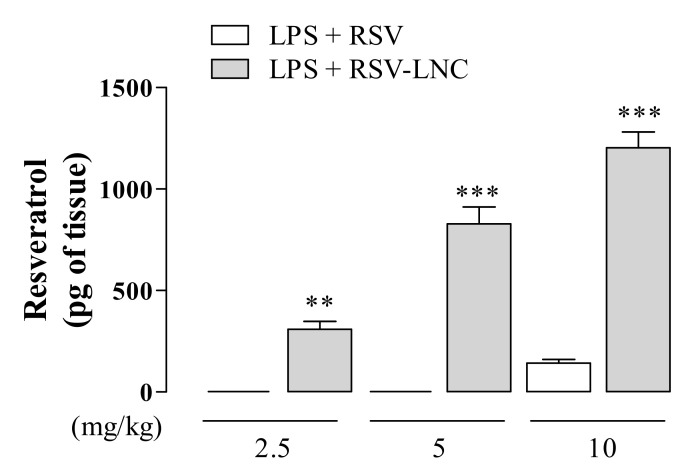
Quantification of RSV in lung tissue after LPS instillation. Mice were orally treated with RSV and RSV-LNCs (2.5, 5, 10 mg/kg) 6 h after intranasal exposure to LPS (25 μg/25 μL). After 24 h of LPS instillation, the lungs were perfused and collected for compound quantification by HPLC analysis. Values represent the mean ± SEM (*n* = 5) of the amount of RSV in lung tissue expressed in pg of the compound per g of tissue. ** *p* < 0.01 and *** *p* < 0.0001 compared to the respective dose of free RSV.

Aiming to evaluate the RSV tissue distribution, we examined the amount of compound in the lungs of mice exposed to LPS treated with nanoencapsulated RSV or in free form. As shown in Figure 6, animals treated with 2.5 or 5 mg/kg of the free molecule presented undetectable levels of RSV, whereas a small amount was observed in animals treated with 10 mg/kg. On the other hand, animals treated with RSV-LNCs at all doses tested presented a significant amount of RSV in lung tissue. Moreover, the detectable amount of lung compound in RSV-LNC-treated mice occurred in a dose-dependent manner.

## 4. Discussion

Since ancient times, natural products have been widely used as an alternative to treat several diseases. Plant-derived extracts and/or compounds isolated from plants are a great source of folk medicines over many centuries [30]. The use of these natural compounds has been shown beneficial effects in several inflammatory diseases such as osteoarthritis, rheumatoid arthritis, and pulmonary inflammatory diseases [31,32,33,34]. RSV is a nonflavonoid polyphenol derived from several plant sources that have a broad spectrum of biological activities, including anti-inflammatory and antioxidant activities [35]. The low bioavailability of RSV, which limits its clinical use, can be overcome by the use of nanocarriers [23]. In this study, we investigated the effect of polymeric nanocapsules loaded with RSV treatment in a situation in which inflammation was already present after LPS exposure in mice. We demonstrated that RSV-LNCs improved lung function, inhibited the influx of leukocytes and pro-inflammatory mediator levels into pulmonary tissue, and reduced lung damage and the expression of transcription factors associated with the inflammatory response. Interestingly, all these effects were accompanied by an increase in antioxidant markers and a reduction in oxidative lung damage. We also observed an increase in RSV levels in pulmonary tissue when mice were treated with RSV-LNCs compared to free RSV, attesting to the improvement in molecular bioavailability.

ALI/ARDS are disorders caused by direct and indirect damage to the lung architecture, which leads to respiratory failure. [36]. ALI/ARDS can be triggered in response to multiple factors, including sepsis and bacterial or viral pneumonia [4,37]. Murine models of ALI/ARDS induced by LPS, a major constituent of Gram-negative bacterial membranes, are widely used as a tool to test potential new therapeutic interventions and to investigate the molecular mechanisms underlying lung injury [38]. Similar to ARDS pathology, several lung injury models have shown that LPS exposure induces neutrophil influx to the alveolar space and lung tissue in mice due to the increased permeability of the alveolar-capillary barrier [23,39]. Similarly, we previously demonstrated that treatment with RSV-LNCs administered 4 h before LPS exposure prevents pathological changes in mice [23]. In the present study, we evaluated the activity of RSV-LNCs after the establishment of the inflammatory process in the lung tissue. We performed the treatment 6h after the administration of LPS based on previous studies. The authors demonstrated that after this period there was an important increase in inflammatory markers, such as an intense influx of leukocytes in the BALF and a large concentration of MPO in the lung tissue of mice. [40,41]. Evaluating our data, we observed that oral treatment with RSV-LNCs, but not RSV in free form, therapeutically reduced neutrophilia in the airway lumen in mice instilled with LPS. Neutrophils play a key role in the progression of ALI/ARDS and are correlated with disease severity. These polymorphonuclear cells, once activated, release their enzyme contents, resulting in an increase in prostanoids and ROS. This process culminates in tissue damage, increased alveolar permeability, and edema. [42]. The decrease in neutrophilia observed in our study is consistent with studies demonstrating that resveratrol treatment can reduce neutrophils in BALF in an ALI mouse model induced by LPS exposure [12,43]. Neutrophil influx and activation generally result in severe damage to lung architecture, which is demonstrated by an abnormal buildup of fluid in the lungs, changes in the alveoli wall, and intense leukocyte migration throughout the lung, noted in histological evaluation [44]. The lower number of neutrophils in the animals treated with nanoencapsulated resveratrol was corroborated by the reduction of MPO, an abundant protease of the granules of these leukocytes. [45,46], and by the improvement of lung damage observed in histological analysis. Similar to our data, Jiang et al. observed that RSV (on free form and higher dose) reduced the elevated MPO pulmonary levels and the pulmonary damage triggered by LPS instillation in mice [43]. Interestingly, we also showed that the amount of RSV that is necessary for the protective effect in LPS-induced lung injury is reduced when loaded in nanocarriers than when loaded in free form.

Expressed by numerous cell types, the phosphatidylinositol 3-kinase (PI3K)/AKT and mitogen-activated protein kinase (MAPK)/extracellular signal-regulated kinase (ERK) pathways are key parts in the course of inflammation, coordinating the gene expression of several proinflammatory cytokines, participating in the body’s immune response [47,48]. Our data show that the decrease in AKT, p38, and ERK phosphorylation occurred together with a reduction in IL-6, TNF-α, MCP-1, MIP-1, MIP-2, RANTES, and KC pulmonary levels. Our results are consistent with another study demonstrating that RSV decreases the production of proinflammatory cytokines by inhibiting the signaling cascades of p38 and ERK in LPS-mediated acute inflammation in rats [49]. RSV also inhibited Akt phosphorylation in RAW264.7 cells stimulated with LPS [50]. Moreover, it is well described that natural molecules such as RSV and curcumin are able to inhibit also other intracellular signaling molecules, such as NF-kB in anti-inflammatory action through multiple signaling pathways [51,52,53,54]. Notoriously, the reduction in all inflammatory parameters associated with RSV-LNC treatment analyzed in our study was accompanied by the improvement in mouse lung function, which was confirmed by improvement in pulmonary elastance.

The involvement of oxidative damage mediated by ROS is likely to be an important event in the development and progression of ARDS pathogenesis. ROS can be secreted by neutrophils, leading to lipid peroxidation [5]. Lipid peroxidation results from an oxidative imbalance in which free radicals deteriorate polyunsaturated fatty acids, by removing electrons and generating cells harmful compounds, such as lipid peroxyl radicals and hydroperoxides [55]. In ALI/ARDS the damage to cell membranes, caused by oxidative stress, allows the influx of liquids into the alveoli, establishing important pulmonary edema. [56]. For research purposes, malondialdehyde or MDA (a by-product of lipid peroxidation) is widely used as a marker of oxidative stress in biological materials [57]. In our study, we observed that posttreatment with RSV-LNCs abolished LPS-induced MDA levels in the lungs, suggesting the improvement of oxidative damage. To neutralize reactive species, cells induce the production and release of antioxidants, such as catalase [5]. Catalases are enzymes responsible for transforming hydrogen peroxide into water and molecular oxygen, preventing lipids oxidation from damaging tissues [58]. We demonstrated here that amelioration of oxidative damage promoted by RSV-LNC treatment occurs together with an increase in catalase pulmonary levels. Our data are supported by studies demonstrating that administration of RSV decreased pulmonary MDA levels and restored catalase activity in an endotoxemia-induced lung injury model [59] and in diabetic rat infarcted brain [60]. It is interesting to note that the decrease in oxidative damage observed in our study had a significant correlation with the increase in Nrf2 expression. Nrf2 is recognized as a crucial transcription factor that mediates protection against oxidant agents. Upon exposure of cells to oxidative stress, this transcription factor translocates into the nucleus to bind antioxidant response elements specific in the promoter regions of its target genes, leading to transcription of antioxidant proteins [61]. Corroborating with our findings, RSV has been shown to decrease oxidative stress in obese asthmatic rat models by inducing the expression of the antioxidant Nrf2 [62]. Moreover, treatment with RSV in human lung epithelial cells exposed to cigarette smoke also inhibited oxidative stress by activating Nrf2 [63].

Despite being the most common, convenient, and extensively used route of drug delivery, oral administration is not ideal for RSV treatment given its low absorption and limited bioavailability [15,64]. Polymeric nanocapsules can protect the drug against destructive organism barriers and increase absorption into the gastrointestinal tract [65,66,67]. PCL is an aliphatic polyester that presents interesting and versatile physicochemical and mechanical properties for drug delivery [68]. In a previous study, we demonstrate that resveratrol-loaded lipid-core nanocapsules increased the concentration of resveratrol in several tissues after daily i.p. or gavage administration [24]. Additionally, lipid-core nanocapsules induced a marked protective effect on the gastrointestinal mucosa compared with the ulcerative effect observed with the free resveratrol. These protective effects could be attributed to a slow release of the polyphenol in the acidic gastric environment of resveratrol-loaded lipid-core nanocapsules compared with free trans-resveratrol [24]. These results are in accordance with the previous work performed by our group, wherein treatment with diclofenac-loaded polymeric nanocapsules and nanospheres or treatment with indomethacin-loaded nanocapsules did not present significant gastrointestinal damage, indicating the slow release of the drugs at the gastric pH [69,70]. Our HPLC data indicate that all beneficial effects observed with RSV-LNC oral treatment in this study occur, at least in part, due to the increase in pulmonary RSV content promoted by the PCL polymeric nanocarrier system. The observed effects may be the result of several factors. Firstly nanoencapsulation leads to an increase in the half-life of lipophilic drugs primarily through gradual and sustained release. Additionally, the delivery of resveratrol into lung tissue promoted by nanocapsules might be due to the enhanced permeability and retention effect triggered by inflammation present in the ARDS model. It is important to note that, significantly higher efficiency was achieved by delivering resveratrol with LNCs.

Given the data obtained in this study, it is clear the pharmacological advantages provided to RSV nanoencapsulation. The oral administration of the RSV nanoformulations allows physical protection of the molecule, decreasing the metabolism of the first hepatic passage and allowing higher quantities of drugs than those when administered in its free form. In the body, greater drug absorption leads to better tissue distribution, according to the physicochemical characteristics of the nanocapsules and physiological conditions, offering an important improvement in the molecule’s effectiveness. Regarding the absence of anti-inflammatory effects of RSV in its free form, it is likely that only sub-pharmacological doses have reached the lung tissue since this molecule has unfavorable kinetics when administered orally. Despite the thought-provoking findings, the mechanisms involved in targeting the nanoparticles are not fully understood and should be further investigated in the future.

## 5. Conclusions

Taken together, all the data reported herein suggest that oral treatment with polymeric nanocapsules containing RSV enhanced lung bioavailability, resulting in an increase in anti-inflammatory and antioxidant effects in a murine lung injury model. We believe also that promisor data showed in our murine pre-clinical study can increment and corroborate with the clinical studies showing the anti-inflammatory and antioxidant effects of RSV intake by humans. The nanostructuring of resveratrol promoted an increase in its bioavailability in the lung tissue, proving to be indispensable to promote the therapeutic effect of low doses of RSV administered orally. Thus, the use of nanosystems of delivery may pave the way for future therapeutic interventions with resveratrol in lung diseases.

## Data Availability

The data presented in this study are available on request from the corresponding author.
